# Parent engagement in children’s eye care behavior and vision-related quality of life: a cross-sectional study

**DOI:** 10.1186/s12889-026-26277-9

**Published:** 2026-02-09

**Authors:** Shu-Mei Liu, Yu-Ting Wang, Jun Chen, Feng Wang, Shu-Fang Shih

**Affiliations:** 1 Zhejiang Philosophy and Social Science Laboratory for Research in Early Development and Childcare, Department of Preschool Education, Jing Hengyi College of Education, Hangzhou Normal University, Hangzhou, China; 2 Shanghai Eye Diseases Prevention & Treatment Center, School of Medicine, Shanghai Eye Hospital, Tongji University, Shanghai, China; 3 Department of Health Policy and Management, School of Public Administration, Hangzhou Normal University, Hangzhou, China; 4 Department of Health Administration, College of Health Professions, 900 E. Leigh Street, Richmond, VA 23298 United States

**Keywords:** Parent engagement, Eye care behavior, Vision-related quality of life, Health Belief Model

## Abstract

**Background:**

Childhood myopia has emerged as a growing public health concern, adversely affecting both visual function and vision-related quality of life (VR-QoL). This study examined the relationships among parent engagement, primary school children’s eye care behaviors, and vision-related quality of life based on the Health Belief Model (HBM).

**Methods:**

In 2022, a total of 2,139 parent–child dyads were recruited from six primary schools in Hangzhou City, China, using stratified cluster sampling. Both children and their parents completed validated, self-administered questionnaires assessing eye care behaviors, engagement efficacy, and HBM variables. Multiple regression analyses were conducted to examine the associations among parent and child health beliefs, parent engagement practices, children’s eye care behaviors, and VR-QoL.

**Results:**

The participated children were aged from 9.24 to 10.19 years old. After adjusting for sociodemographic factors, parents with greater eye care knowledge, fewer perceived barriers, and stronger engagement efficacy were more likely to support their children’s eye care behaviors. Among children, higher levels of eye care knowledge, perceived severity of myopia, and perceived benefits of protective practices were significantly associated with more frequent engagement in eye care behaviors. Children with stronger perceptions of severity and benefits, lower susceptibility, fewer barriers, and better eye care behavior reported higher VR-QoL.

**Conclusions:**

Health belief variables were significantly associated with parent engagement, children’s eye care behaviors, and VR-QoL. These findings highlight the importance of considering both parent- and child-level cognitive and behavioral factors when examining childhood eye health. Theory-informed assessments can inform the development of contextually appropriate vision health promotion strategies.

**Supplementary Information:**

The online version contains supplementary material available at 10.1186/s12889-026-26277-9.

## Background

Vision impairment and blindness are pressing global public health challenges, affecting over 2.2 billion individuals worldwide. Notably, over one billion cases involve vision impairments that are either preventable or untreated, contributing to significant social and economic burdens [1]. Among these issues, childhood myopia has emerged as a growing public health crisis, particularly in East Asian countries [2]. China has been experiencing a significant and potentially worsening epidemic of childhood myopia over the past few decades [3]. In China, the prevalence of myopia among children and adolescents reached 54.7% in 2021, exacerbated by the COVID-19 pandemic [4]. This was because children were confined to home environments, engaging in prolonged screen-based learning while outdoor activities were significantly reduced. In 2022, the overall prevalence of myopia among children and adolescents in China reached 51.9%, with 36.7% of elementary school students affected [5]. Previous studies have shown that children aged 6–10 years are particularly vulnerable to developing myopia, with the majority of cases occurring among students in grades three and four of elementary school [6, 7].

Early-onset myopia is particularly concerning as it is associated with a heightened risk of progression to high myopia, which can lead to irreversible vision loss [8]. Vision impairment during childhood has far-reaching consequences, potentially disrupting motor, language, emotional, social, and cognitive development [1]. These issues affect not only individual development but also have broader implications for societal well-being and public health [9]. Moreover, myopia negatively impacts children’s vision-related quality of life (VR-QoL) [10] and may also diminish the overall quality of life of both children and their families [11]. More severe myopia is associated with a greater decline in VR-QoL for both children and their parents, particularly among those with high myopia [11].

Preventive eye care behavior can mitigate the onset and progression of myopia and alleviate symptoms such as asthenopia [2]. Established risk factors for myopia include prolonged near-work activities, excessive screen time, and limited time spent outdoors [12, 13]. On the other hand, protective behaviors, such as engaging in outdoor activities, maintaining appropriate working distances, and taking regular breaks from near work every 30 min, have demonstrated beneficial effects [14]. Specific outdoor exposure, such as at least 15 min in environments with sunlight levels of 2,000 lx or more, has also been shown to be protective [15, 16]. Furthermore, the absence of routine vision screenings has been significantly associated with myopia progression [17]. Despite the known benefits of preventive eye care, many children do not consistently engage in protective practices. For example, a systematic review found that children aged 6 to 14 in China reported an average daily screen time of 2.77 hours, with 46.4% exceeding two hours per day [18]. Similarly, a recent study indicate that one in five children in China spend less than one hour outdoors daily [19]. In addition, the prevalence of poor myopia-related behaviors among children ranged from 23.44% to 84.82%, with incorrect sitting posture being particularly common [20]. These findings suggest that children’s eye care behaviors remain suboptimal and highlight the need for further intervention and reinforcement [21].

The family environment plays a vital role in shaping children’s eye health behaviors. Parental involvement, in particular, has a significant influence on children’s daily routines including their screen use and engagement in near work or outdoor activities [22]. Parents who perceive a higher risk of myopia and exhibit stronger confidence in their ability to manage their children’s behavior are more likely to actively support healthy eye care practices [23]. Previous studies have shown that parental rules limiting screen time are effective in reducing children’s screen use, while restrictions on outdoor play may inadvertently increase sedentary behaviors such as television viewing [24, 25]. Furthermore, an intervention study has shown that parent involvement in eye health programs can positively influence children’s eye care behavior, even at the preschool level [26]. However, studies in China have shown that parents often lack awareness of the adverse consequences of myopia, possess limited knowledge of preventive measures, and demonstrate inadequate practices related to myopia prevention and control [27–29]. Therefore, future efforts should prioritize enhancing parental knowledge, raising awareness, and promoting active involvement in children’s myopia management.

The Health Belief Model (HBM) is a widely recognized theoretical framework for understanding health-related behaviors, including those related to eye care [30, 31]. Originally conceptualised by Becker in 1974 and later refined by Rosenstock in 1990, the HBM posits that individuals’ engagement in health behaviors is influenced by their personal beliefs about health risks and outcomes [32]. The model includes six constructs: perceived susceptibility, perceived severity, perceived benefits, perceived barriers, self-efficacy, and health behavior itself. While the first four constructs formed the model’s original foundation, the addition of self-efficacy has enhanced its explanatory power and predictive validity [32].

Understanding and addressing the factors that influence children’s eye care behavior is essential for the prevention and control of childhood myopia. Theory-based approaches provide a robust foundation for the design of behavior change interventions, allowing for the development of strategies that target specific determinants [26, 33]. However, the relationships among health belief constructs, parent engagement, children’s eye care behaviors, and VR-QoL remain insufficiently explored. Although prior research has linked health belief constructs to health-related quality of life (HRQoL) and demonstrated the effectiveness of theory-informed interventions in improving outcomes [34, 35], more empirical evidence is needed to understand their associations.

Accordingly, the present study employed HBM as the framework to investigate the associations among parent engagement, children’s eye care behavior, health belief constructs, and vision-related quality of life. The findings can inform the design of targeted interventions that promote effective parent engagement and foster sustainable improvements in children’s vision health and well-being.

## Methods

### Study design

A cross-sectional study was conducted over a two-month period between April and May 2022. The study targeted third and fourth-grade students and their parents from primary schools in Hangzhou City, Zhejiang Province, China. We selected only third- and fourth-grade students for this study because previous research in China has shown that the prevalence of myopia is increasing most rapidly among children aged 6–10 years [6, 7]. Children within this age range are typically enrolled in the third- and fourth-grades, making this population highly relevant to our study’s focus on children’s eye care behaviors.

According to the Hangzhou Education Bureau (2022), approximately 700,000 students were enrolled in 507 primary schools across the city [36]. We employed a stratified cluster sampling method to recruit participants. First, three districts were randomly selected from the 10 municipal districts in Hangzhou city, including a main urban district, a non-main urban district, and an urban-rural fringe district. We randomly selected six primary schools from these three distinct areas. The sample size within each district was proportionally allocated based on student population. Sample size estimation was performed using G*Power 3.1.9.4. Assuming a statistical power of 0.80, a two-tailed α of 0.05, and a large effect size (f² = 0.80), the minimum sample sizes required for the three regression models (containing 11, 12, and 12 independent variables, respectively, including both core and control variables) were each estimated to be fewer than 800 participants. Based on prior field experience, we anticipated a nonresponse or attrition rate of approximately 20%. Using the formula “minimum sample size/response rate,” we determined that at least 1,000 questionnaires would need to be distributed to meet power requirements. We distributed 2,376 questionnaires to third- and fourth-grade students and their parents, exceeding the required sample size and ensuring sufficient statistical power for all analyses. The response rates were 94.9% for children and 97.6% for parents.

### Participants and procedures

Consent forms were distributed through students to their parents and returned with signatures from both parties. Students also provided assent. After receiving consent, the student surveys were administered in person at the school. Parents completed their questionnaires online using Wen Juanxing (https://www.wjx.cn/), a widely used Chinese survey platform. Survey links were distributed via WeChat, China’s most popular social messaging application.

After excluding incomplete responses and those completed by individuals other than the parents (e.g., grandparents or other relatives), 2,139 valid parent–child dyads were retained for the final analysis.

## Measures

Questionnaires were developed based on established literature [30, 37, 38] (see Supplemental Information for key questions used in this study). To assess its content validity, a review panel composed of six experts was convened, including ophthalmologists, school medical staff, and scholars specializing in myopia prevention and control, health education, and child health. All items achieved a Content Validity Index (CVI) scores higher than 0.80. Revisions and refinements were made to the questionnaire in accordance with the experts’ comments and suggestions. Additionally, a pre-test was conducted on 30 parent-child dyads, which verified that the scale was appropriately applicable for the target study population.

In terms of instrument, this study followed established guidelines for cross-cultural scale translation and adaptation [39, 40]. Initially, two scholars with expertise in psychology and education independently conducted forward translations of the instrument. An expert panel, comprising professionals in education and linguistics, then reviewed the translations to ensure semantic accuracy and cultural relevance. A third-party translator subsequently performed a blind back-translation, which was compared with the original version to identify and resolve any discrepancies.

### Children’s vision-related quality of life (VR-QoL)

Children’s VR-QoL was assessed using 10 items. Example statements included: “I experience eye fatigue, dryness, or soreness,” and “My vision problems hinder my ability to participate in sports or physical activities.” Responses were rated on a 4-point Likert scale from “always” (1) to “never” (4), with higher scores reflecting better VR-QoL. The internal consistency of this scale was high (Cronbach’s α = 0.86).

### Children’s eye care behavior

Seventeen items were used to assess children’s eye care practices, covering four domains: outdoor activity (3 items), near work activity (8 items), sleep (4 items), and eye examinations (2 items). Children reported the frequency of behaviors over the past six months. Sample items included: “On weekends or holidays, I spent more than 2 hours outdoors,” and “I took a 10-minute break after every 30 minutes of reading or writing.” Items were rated on a 4-point Likert scale ranging from “never” (1) to “always” (4), with higher scores indicating more frequent adoption of healthy eye care behaviors (Cronbach’s α = 0.89).

### Parent engagement of children’s eye care behavior

Parent engagement was measured using 15 items spanning outdoor activity (2 items), near work behavior (8 items), sleep habits (3 items), and eye examinations (2 items). Parents reported the frequency of their involvement over the past six months. Sample items included: “I encourage my child to take breaks after 30–40 minutes of screen-based learning,” and “I restrict my child from reading or using electronic devices while walking or riding in a vehicle.” Responses were rated on a 5-point Likert scale from “never” (1) to “always” (5), with higher scores indicating greater parent engagement (Cronbach’s α = 0.90).

### Parent engagement efficacy

Parents’ confidence in managing their children’s eye care behavior was assessed using a 15-item scale covering the same four behavioral domains. Sample items included: “I can ensure my child spends more than two hours outdoors on weekends,” and “I can arrange regular eye examinations for my child every six months.” Responses were rated on a 5-point Likert scale from “not confident at all” (1) to “completely confident” (5), with higher scores reflecting greater engagement efficacy (Cronbach’s α = 0.91).

### Health belief model constructs

A structured questionnaire based on HBM was developed to assess health beliefs related to eye care among both children and parents [30, 38]. The child version included 11 items, and the parent version included 13 items, categorized into four subscales:

1. Perceived Susceptibility (2 items): e.g., “I believe that I/my child is prone to developing myopia.”
2. Perceived Severity (2 items): e.g., “If I/my child develops myopia at an early age, it is more likely to progress to high myopia.”
3. Perceived Benefits (2 items): e.g., “Developing good eye care habits can prevent or slow the progression of myopia.”
4. Perceived Barriers (7 items): e.g., “I or my child have too much homework, leaving no time for outdoor activities.”

Children responded using a 4-point Likert scale (“strongly disagree” = 1 to “strongly agree” = 4), with Cronbach’s alpha values of 0.60 (susceptibility), 0.60 (severity), 0.73 (benefits), and 0.60 (barriers). Parents used a 5-point scale (“strongly disagree” = 1 to “strongly agree” = 5), with alpha values of 0.66, 0.65, 0.90, and 0.80, respectively.

### Eye care knowledge

Eye care knowledge was measured with 12 items across three domains: myopia risk (3 items), behavioral factors (6 items), and regular eye examination (3 items). Participants selected one response per item: “correct,” “incorrect,” or “don’t know.” Sample items included: “Myopia is a disease,” and “If I/my child has normal vision, regular eye examinations are not necessary.” Correct responses were scored as 1, and all others as 0. Higher total scores indicated greater knowledge. Kuder-Richardson reliability coefficients were 0.67 for children and 0.60 for parents.

## Sociodemographic characteristics

### Parental characteristics

Collected parental variables included parent survey respondent (mother or father), age (≤ 35 vs. >35 years), educational qualification (college or higher vs. high school or lower), and household income (not low vs. low), with low income defined according to the local government’s subsidy standard [41]. Parents also reported their child’s vision status (non-myopia vs. myopia), based on school-issued vision health reports.

### Children characteristics

Child-level variables included sex (girl or boy), primary caregiver (mother, father, or grandparents), cohabitation with parents (yes or no), and ownership of electronic devices (yes or no).

### Statistical analysis

Descriptive statistical analyses were conducted for all study variables. For categorical variables, frequencies and percentages were reported. These included parent-reported variables (parent survey respondent, age, education, household income, and child’s vision status) and child-reported variables (sex, primary caregiver, cohabitation with parents, and electronic device ownership). For continuous variables, means and standard deviations were calculated. These included child-reported measures (eye care knowledge, eye care health beliefs, eye care behaviors, and vision-related quality of life) and parent-reported measures (eye care knowledge, eye care health beliefs, engagement efficacy, and engagement in the child’s eye care behaviors). Multiple linear regression analyses were conducted to assess the associations among parent engagement, child eye care behaviors, and VR-QoL, adjusting for relevant sociodemographic variables. Statistical significance was set at *p*<.05. All statistical analyses were performed using SPSS version 26.0 (IBM Corp., Armonk, NY, USA).

### Ethical considerations

Written informed consent and assent was obtained from parents and children, respectively, affirming their voluntary participation. Ethical approval was granted by the Institutional Review Board at Hangzhou Normal University (Approval No.: 2021030801).

## Results

### Descriptive statistics

In the present study, third-grade students ranged in age from 7.75 to 11.42 years (Mean = 9.24), and fourth-grade students ranged from 7.50 to 11.75 years (Mean = 10.19). Among the 2,139 participating children, 1,113 were boys (52.0%) and 1,026 were girls (48.0%). According to parental reports, 557 children (26.0%) had been diagnosed with myopia. Additionally, 12.5% of children did not cohabit with either parent, and 73.2% owned personal electronic devices (Table 1).

**Table 1:** Descriptive statistics of participants

Variables	*n*	%
Total	2,139	100.0
Parent survey respondent		
Mother	1,692	79.1
Father	447	20.9
Age		
≤ 35 years	720	33.7
> 35 years	1,419	66.3
Educational qualification		
College or higher	1,451	67.8
High school or lower	688	32.2
Household income		
Not low	1,035	48.4
Low	1,104	51.6
Child’s vision status		
Non-myopia	1,582	74.0
Myopia	557	26.0
Child sex		
Girl	1,026	48.0
Boy	1113	52.0
Primary caregiver		
Mother	1,470	68.7
Father	255	11.9
Grandparents	414	19.4
Cohabitation with parents		
Yes	1,872	87.5
No	267	12.5
Electronic device ownership		
No	573	26.8
Yes	1,566	73.2

Parent–child dyads *N* = 2,139

Children reported relatively high levels of eye care behavior (Mean = 3.15, SD = 0.63; range: 1–4), perceived benefits (Mean = 3.50, SD = 0.90), and vision-related quality of life (VR-QoL) (Mean = 3.49, SD = 0.55) (Table2).

**Table 2:** Descriptive statistics of key variables

Variables				Items	Score			Mean		SD
**Child**										
Eye care knowledge				12	0–1			0.61		0.19
Eye care health beliefs										
Perceived susceptibility				2	1–4			2.30		0.94
Perceived severity				2	1–4			2.88		0.98
Perceived benefits				2	1–4			3.50		0.90
Perceived barriers				5	1–4			1.78		0.66
Eye care behavior				17	1–4			3.15		0.63
Outdoor activity				3	1–4			3.12		0.78
Eye use habits				8	1–4			3.18		0.70
Sleep				4	1–4			3.13		0.76
Eye examinations				2	1–4			3.17		0.87
Vision-related quality of life				10	1–4			3.49		0.55
**Parent**										
Eye care knowledge				12	0–1			0.81		0.14
Eye care health beliefs										
Perceived susceptibility				2	1–5			3.38		0.92
Perceived severity				2	1–5			3.70		0.98
Perceived benefits				2	1–5			4.45		0.85
Perceived barriers				7	1–5			2.74		0.80
Engagement efficacy				15	1–5			3.83		0.67
Engagement related to child’s eye care behavior				15	1–5			3.70		0.63

Parent–child dyad *N* = 2,139

*SD * standard deviation

The majority of parent respondents were mothers (79.1%, *n* = 1,692) while 20.9% were fathers (*n* = 447). Most parents were over 35 years old (66.3%), had attained at least a college-level education (67.8%), and 51.6% reported low-income household status. The majority of children’s primary caregivers were mothers (68.7%) (Table 1).

Parents have high levels of eye care knowledge (Mean = 0.81, SD = 0.14), perceived severity (Mean = 3.70, SD = 0.98), perceived benefits (Mean = 4.45, SD = 0.85), parent engagement efficacy (Mean = 3.83, SD = 0.67), and actual engagement in children’s eye care (Mean = 3.70, SD = 0.63) (Table 2).

### Factors associated with parent engagement

Multiple regression analysis identified several significant predictors of parent engagement in children’s eye care behavior. Parents from higher-income households were less likely to engage (β =−0.04, SE = 0.02, *p* =0.007), whereas those with greater eye care knowledge (β = 0.03, SE = 0.07, *p* =0.036), fewer perceived barriers (β =−0.10, SE = 0.01, *p* <0.001), and higher engagement efficacy (β = 0.72, SE = 0.02, *p* <0.001) demonstrated significantly greater engagement in their children’s eye care (Table 3).

**Table 3:** Factotrs associated with parent engagement

Variables	Parent engagement		
	* β *	SE	*P*
Intercept	1.21	0.09	< 0.001***
Parent (ref: mother)			
Father	−0.01	0.02	0.423
Age (ref: ≤ 35 years)			
> 35 years	−0.01	0.02	0.453
Educational qualification(ref: college or higher)			
High school or lower	−0.03	0.02	0.107
Household income(ref: not low income)			
Low income	−0.04	0.02	0.007**
Child’s vision status(ref: non-myopia)			
Myopia	0.03	0.02	0.085
Eye care knowledge	0.03	0.07	0.036*
Eye care health beliefs			
Perceived susceptibility	0.03	0.01	0.051
Perceived severity	0.00	0.01	0.890
Perceived benefits	−0.01	0.01	0.459
Perceived barriers	−0.10	0.01	< 0.001***
Parent engagement efficacy	0.72	0.02	< 0.001***

Parent–child dyad *N* = 2,139

*SE* standard error

*: <0.05: **:<0.01; ***<0.001

### Factors associated with children’s eye care behavior

Multiple regression analysis revealed that boys (β =−0.10, SE = 0.03, *p* <0.001), children cared for by grandparents (β =−0.05, SE = 0.03, *p* =0.013), and those owning personal electronic devices (β=−0.08, SE = 0.03, *p* <0.001) exhibited lower levels of eye care behavior. On the other hand, greater eye care knowledge (β = 0.13, SE = 0.07, *p* <0.001), higher perceived severity (β = 0.06, SE = 0.02, *p* =0.009), perceived benefits (β = 0.07, SE = 0.02, *p* =0.001), and stronger parent engagement (β = 0.07, SE = 0.02, *p* =0.001) were positively associated with children’s eye care behavior (Table 4).

**Table 4:** Factors associated with children’s eye care behavior

Variables	Children’s eye care behavior		
	*β*	SE	*p*
Intercept	3.11	0.10	< 0.001***
Child gender (ref: girl)			
Boy	−0.10	0.03	< 0.001***
Primary caregiver (ref: mother)			
Father	−0.02	0.04	0.411
Ggrandparents	−0.05	0.03	0.013*
Cohabitation with parents (ref: yes)			
No	−0.02	0.04	0.256
Electronic device ownership (ref: no)			
Yes	−0.08	0.03	< 0.001***
Eye care knowledge	0.13	0.07	< 0.001***
Eye care health beliefs			
Perceived susceptibility	−0.04	0.02	0.072
Perceived severity	0.06	0.02	0.009**
Perceived benefits	0.07	0.02	0.001**
Perceived barriers	−0.30	0.02	< 0.001***
Household income (ref: not low income)			
Low income	0.01	0.03	0.617
Child’s vision status (ref: non-myopia)			
Myopia	−0.02	0.03	0.432
Parent engagement related to children’s eye care behavior	0.07	0.02	0.001***

Parent–child dyad *N* = 2,139

*SE* standard error

*: <0.05: **:<0.01; ***<0.001

### Factors associated with children’s VR-QoL

Children with myopia (β=−0.22, SE = 0.03, *p* <0.001), those from lower-income households (β=−0.05, SE = 0.02, *p* =0.007), and those who owned personal electronic devices (β=−0.06, SE = 0.02, *p* =0.001) were more likely to report lower VR-QoL. In contrast, higher perceived severity (β = 0.07, SE = 0.01, *p* =0.001), perceived benefits (β = 0.15, SE = 0.01, *p* <.001), lower perceived susceptibility (β = −0.15, SE = 0.01, *p* <0.001), lower perceived barriers (β=−0.20, SE = 0.02, *p* <0.001), and greater eye care behavior (β = 0.09, SE = 0.02, *p* <0.001) were significantly associated with better VR-QoL (Table 5).

**Table 5:** Factors associated with children’s vision-related quality of life (VR-QoL)

Variables	Children’s vision-related quality of life		
	*β *	SE	*p*
Intercept	3.55	0.11	< 0.001***
Child sex (ref: girl)			
Boy	0.00	0.02	0.996
Primary caregiver (ref: mother)			
Father	−0.03	0.03	0.132
Grandparents	0.01	0.03	0.648
Cohabitation with parents (ref: yes)			
No	−0.03	0.03	0.135
Electronic device ownership (ref: No)			
Yes	−0.06	0.02	0.001**
Eye care health beliefs			
Perceived susceptibility	−0.15	0.01	< 0.001***
Perceived severity	0.07	0.01	0.001**
Perceived benefits	0.15	0.01	< 0.001***
Perceived barriers	−0.20	0.02	< 0.001***
Children’s eye care behavior	0.09	0.02	< 0.001***
Household income (ref: not low income)			
Low income	−0.05	0.02	0.007**
Child’s vision status (ref: non-myopia)			
Myopia	−0.22	0.03	< 0.001***
Parent engagement related to children’s eye care behavior	−0.01	0.02	0.550

Parent–child dyad *N* = 2,139

*SE* standard error

*: <0.05: **:<0.01; ***<0.001

## Discussion

This study highlights the pivotal role of parents in shaping children’s eye care. Our findings demonstrate that parents with higher levels of eye care knowledge, fewer perceived barriers, and stronger parent engagement efficacy were more likely to implement positive parent engagement strategies in their children’s eye care. These results are consistent with prior research indicating that parental barriers such as misconceptions about vision status (e.g., assuming the child has normal vision), perceived lack of necessity for eye examinations, child non-cooperation, and limited time or access to care can hinder effective engagement [30, 42]. Moreover, studies show that higher parental health literacy and confidence are key enablers of proactive practices, particularly in managing children’s screen time and encouraging preventive eye care behavior [43]. Other investigations emphasize the need to understand parents’ perspectives on vision health and healthcare navigation to improve service utilization and awareness of screening programs [44–46]. These insights collectively suggest that interventions should prioritize enhancing parental knowledge, self-efficacy, and awareness of eye care resources to effectively support children’s vision health.

In addition to parental factors, children’s health beliefs and demographic factors were also significantly associated with their eye care practices. This study found that girls, children without personal electronic devices, and those primarily cared for by their mothers demonstrated higher levels of eye care knowledge, perceived severity, perceived benefits, and lower perceived barriers. These characteristics were positively associated with more frequent engagement in healthy eye care behaviors. These findings also align with previous research showing that children with greater awareness of myopia severity and benefits of prevention, who also have fewer perceived barriers, are more likely to practice protective behaviors [30]. Furthermore, evidence from review studies suggests that spending more than two hours outdoors daily may be effective in delaying the onset and progression of myopia in children [47, 48]. However, our findings highlight gaps in achieving this goal. Our study shows that nearly half of the children in this study reported inadequate outdoor activity on weekends or holidays, and over one-third had not undergone biannual eye examinations. These findings emphasize the urgent need for school-based eye care education programs that not only engage children directly but also work in tandem with parents to address knowledge gaps, elevate perceptions of risk and benefits, and lower structural and behavioral barriers to myopia prevention [49].

Children’s health beliefs were also associated with their VR-QoL. Specifically, children who reported higher perceived severity and benefits, and lower perceived susceptibility and barriers, demonstrated better VR-QoL. Additionally, there is a significant disparity in VR-QoL between children with and without myopia, reinforcing prior research that links vision problems to lower quality of life [10, 50]. For example, students with myopia reported significantly lower total VR-QoL scores than their peers with normal vision [51]. Children with visual impairments are also at increased risk of psychological challenges and social withdrawal [52]. Moreover, positive eye care behaviors were a protective factor for VR-QoL, mirroring findings that good habits such as regular outdoor activity and vision checks, are positively associated with quality of life [51]. These results suggest that school-based eye health initiatives should extend beyond screenings to include behavioral components and emotional well-being supports that promote holistic vision health in children.

Our findings also underscore the pivotal role of parents in promoting children’s eye health and reinforce the utility of health belief constructs in guiding eye care interventions. Parents with greater eye care knowledge, fewer perceived barriers, and stronger engagement efficacy were more likely to support their children’s healthy eye care behavior. These results align with previous research identifying knowledge, confidence, and lower perceived obstacles as key facilitators of parental involvement [30, 43]. Moreover, prior research has emphasised that parenting plays a critical role in shaping children’s eye care behavior [23]. Despite this, over one-third of parents reported low levels of involvement in key preventive behaviors such as scheduling outdoor activities or vision screenings. These findings highlight the need for school-based and family-centred interventions to strengthen parent education and support, particularly around the importance of early intervention and consistent monitoring [42, 44–46].

The widespread ownership of electronic devices among children (73%) underscores the need for enhanced parental guidance and targeted health education regarding screen use, particularly given its established association with increased screen time, reduced outdoor activity, and elevated risk of myopia progression [53, 54]. Research has shown that smartphone overuse is a risk factor for paediatric dry eye disease and may exacerbate digital eye strain and myopia, particularly among younger children [55]. This concern became more pronounced during the COVID-19 pandemic, as prolonged home confinement and increased reliance on online learning contributed to accelerated myopia progression in children [56]. A recent meta-analysis further demonstrated that each additional hour of daily screen time is associated with a 21% increased risk of developing myopia in children and adolescents [57]. Another systematic review confirmed that near work, including prolonged screen use, is significantly associated with myopia development in children [58]. Beyond clinical impacts, children and adolescents themselves report awareness of visual and behavioral consequences of excessive media device use [59], while shared screen viewing and parent-child conversation may buffer some negative effects [60]. Importantly, parental monitoring has demonstrated a protective effect, prospective studies show that children whose media use is actively monitored by parents are more likely to adopt healthier screen-time behaviors [61]. Both national and international guidelines underscore the importance of limiting screen exposure. For example, the American Academy of Pediatrics recommends no more than one hour per day of high-quality programming for children aged 2 to 5, and consistent limits for older children [62]. Similarly, Australian health authorities emphasise limiting recreational screen time to less than two hours per day for children and adolescents, advocating for frequent breaks and more time spent outdoors to support healthy development and visual health [63]. These recommendations offer actionable strategies that can be reinforced through coordinated school and family-based interventions.

Vision screening, particularly in early childhood, remains a critical tool for identifying vision issues before they impact academic performance and social development [64]. However, inconsistencies in school-based screening practices persist globally, with many programs failing to address behavioral factors associated with myopia progression such as prolonged screen time and reduced outdoor activity [65, 66]. Recent research shows that screen time significantly increases the risk of myopia, and that visual acuity can decline with increased device use [57, 67]. For instance, a meta-analysis found that each additional hour of screen time was associated with a 21% higher risk of developing myopia [67]. Another study found a notable increase in screen time and corresponding deterioration in children’s vision during COVID-19-related school closures [68]. To respond to these trends, School-based vision health programs should integrate both visual acuity assessments and brief evaluations of digital media habits, near work behaviors, and time spent outdoors both during and outside of the school year, including periods such as summer and winter breaks. Although implementation research in this area is still developing, applying an implementation science lens can enhance the adoption, integration, and sustainability of school-based vision health programs. Embedding parent and child questionnaires into routine screening protocols not only supports early identification of high-risk profiles but also facilitates data collection that informs tailored, context-specific health promotion strategies. By building a school health system that functions as a learning health system, one that systematically collects, integrates, and analyzes data to generate actionable insights, schools can continuously refine and adapt their vision care interventions. Addressing both clinical and behavioral determinants, such as screen-related habits, through classroom-based curricula and family engagement, further supports sustained behavior change.

It is equally important to address the broader social determinants of health that shape disparities in eye care outcomes. Lower-income families often face barriers such as limited access to affordable vision care, reduced health literacy, and fewer opportunities for health-promoting behaviors [69, 70]. These structural inequities contribute to disparities in both myopia prevention and treatment. For instance, sight-threatening ocular conditions are more likely to go undiagnosed among children from less affluent households [71]. By incorporating social determinants of health into intervention design, vision health strategies can be more effectively tailored to meet the needs of disadvantaged families and reduce long-standing inequities.

To address these multifaceted challenges, coordinated partnerships across sectors are essential. Schools, healthcare providers, public health agencies, and community organisations should collaborate to develop and implement comprehensive vision health strategies [72]. These partnerships can support school-based programmes by facilitating access to screening, delivering culturally tailored health education, and addressing environmental and behavioral risks for childhood myopia. Strengthening multisectoral collaboration is vital to reduce disparities and ensure all children receive the support needed for optimal eye health and VR-QoL.

This study has several limitations. First, owing to the cross-sectional design, causal relationships among variables cannot be established. Longitudinal and interventional studies are needed to confirm the directional pathways proposed and to assess the sustained impact of parental engagement and behavioral interventions on children’s vision health. Second, all data were collected through self-reported questionnaires completed by parents and children, which introduces the potential for two types of bias. Recall bias may arise from retrospective questions, such as estimating “outdoor activity duration in the past month,” as participants may struggle to accurately recall past behaviors. Social desirability bias is also possible, as some respondents may provide answers that align with perceived health norms rather than their actual behaviors. While the study employed validated instruments with acceptable to high reliability (Cronbach’s alpha ranging from 0.60 to 0.98), future research could be strengthened by incorporating observational or clinical validation methods. For example, objective tools such as activity trackers could be used to supplement self-reported data and reduce the influence of these biases. Third, children’s myopia status was reported by parents based on school-issued vision reports, which may have limited diagnostic accuracy. To mitigate this bias, parents are advised to incorporate objective data from recent school-based eye examinations and physician diagnoses, which are communicated via mobile phone notifications. Nevertheless, clinical assessments by eye care professionals would provide more robust validation.

Fourth, the study focused on a single metropolitan area in China, which may limit the generalisability of findings to other regions or countries. Variations in cultural norms, healthcare infrastructure, and digital access may influence parent engagement and children’s eye care behavior differently in other settings. In addition, because our sample was restricted to third- and fourth-grade students from selected districts, the results may not reflect the experiences or behaviors of younger or older children, or those from different geographic or socio-cultural backgrounds. To strengthen external validity and generalizability, future research should expand the sample to include additional grade levels and a wider range of schools across diverse regions.

Moreover, while several dimensions of the HBM and factors related to social determinants were incorporated into the study, future research could explore additional contextual factors such as access to paediatric ophthalmologists, environmental exposures such as indoor lighting or air quality, and other locally relevant structural barriers.

This study underscores the critical role of parent engagement and child health beliefs in shaping children’s eye care behavior and VR-QoL. The findings suggest that both parent- and child-level factors, such as eye care knowledge, perceived barriers, and self-efficacy, should be central to the design of future interventions. Integrating educational, behavioral, and screening components into school-based programmes offers a promising strategy to delay myopia onset and support lifelong vision health.

Given the rising prevalence of childhood myopia and the increasing use of screen-based technologies at home and in schools, targeted, theory-informed interventions are urgently needed. These strategies must also be tailored to address disparities affecting socioeconomically disadvantaged families, where gaps in health literacy and access to care are more pronounced.

Future research should explore the effectiveness of multi-tiered interventions that combine school-based education, mobile health tools, and policy-level initiatives aimed at reducing screen exposure and expanding access to paediatric vision screening. Additionally, incorporating social determinants of health into programme design can help identify and reduce inequities in vision care. Cross-cultural research can provide deeper insight into how different structural, social, and environmental conditions shape children’s vision outcomes globally. Collaborative, multisectoral partnerships are essential for developing sustainable, equity-focused strategies that ensure all children, regardless of background, achieve optimal eye health and VR-QoL.

## Supplementary Information

Supplementary Material 1

## Data Availability

The datasets generated and analyzed during the current study are not publicly available due to the informed consent provided by participants, which specifies that their data will be used solely for the purposes of this study and handled anonymously. However, the datasets may be available from the corresponding author upon reasonable request.

## Abbreviations

HBM: Health Belief Model
SD: Standard deviation
SE: Standard error
VR-QoL: Vision-related quality of life
