# Supplementary material for: Parent engagement in children’s eye care behavior and vision-related quality of life: a cross-sectional study
Source: BMC Public Health. 2026 Feb 9;26:879. doi: 10.1186/s12889-026-26277-9 (PMC12983705; doi:10.1186/s12889-026-26277-9)
Supplement: Supplementary file 1 — Supplementary Material 1 [file 12889_2026_26277_MOESM1_ESM.docx]

| **Table S-1** Variables of Children’s vision-related quality of life | | | | |
| --- | --- | --- | --- | --- |
| Questions | (1) Always | (2) Sometimes | (3) Seldom | (4) Never |
| 1. I experience eye fatigue, dryness or soreness. |  |  |  |  |
| 2. After using my eyes at close range for a long time, I feel blurred vision. |  |  |  |  |
| 3. I have difficulty seeing the words on the blackboard when sitting at the back of the classroom. |  |  |  |  |
| 4. I have the phenomenon of squinting to see things or leaning close to the screen. |  |  |  |  |
| 5. My vision problems hinder my ability to participate in sports or physical activities. |  |  |  |  |
| 6. My vision problem affects my academic performance. |  |  |  |  |
| 7. My vision problem affects getting along with classmates or friends. |  |  |  |  |
| 8. Because of my vision problems, I can't keep up with other peers in school activities. |  |  |  |  |
| 9. I feel worried, nervous or depressed about my vision condition. |  |  |  |  |
| 10. I worry that my vision problems will affect my future study and work. |  |  |  |  |

| **Table S-2.** Variables of Children’s eye care behavior | | | | |
| --- | --- | --- | --- | --- |
| Questions | (1) Never | (2) Seldom | (3) Sometimes | (4) Always |
| 1. On school days, I have outdoor activities for more than 1 hour every day after school and before dark. |  |  |  |  |
| 2. On weekends or holidays, I spent more than 2 hours outdoors. |  |  |  |  |
| 3. During every class break, I go to the corridor outside the classroom or have outdoor activities. |  |  |  |  |
| 4. I took 10-minute break after every 30 minutes of reading or writing. |  |  |  |  |
| 5. After studying using an electronic screen for 30 - 40 minutes, I go outdoors or look into the distance for at least 10 minutes. |  |  |  |  |
| 6. When I play games/watch videos/watch movies on electronic products, each time lasts no more than 15 minutes. |  |  |  |  |
| 7. When I play games/watch videos/watch movies on electronic products, the total daily time is no more than 1 hour. |  |  |  |  |
| 8. When I read or do homework, I keep a distance of more than 35 centimeters. |  |  |  |  |
| 9. I have a correct posture when reading, writing or using electronic products. |  |  |  |  |
| 10. I read, do homework or use electronic products in sufficient light. |  |  |  |  |
| 11. I don't read books or use electronic products while walking or taking a vehicle. |  |  |  |  |
| 12. I sleep at least 10 hours every day. |  |  |  |  |
| 13. I go to bed before 9 o'clock every day. |  |  |  |  |
| 14. I don't use electronic products 1 hour before going to bed every day. |  |  |  |  |
| 15. On school days, I take a noon break for at least half an hour at school every day. |  |  |  |  |
| 16. I have my vision checked by an ophthalmologist regularly every six months. |  |  |  |  |
| 17. After each school vision check, I look at my vision check report sheet. |  |  |  |  |
